# GEP Tree-Based Prediction Model for Interfacial Bond Strength of Externally Bonded FRP Laminates on Grooves with Concrete Prism

**DOI:** 10.3390/polym14102016

**Published:** 2022-05-16

**Authors:** Muhammad Nasir Amin, Mudassir Iqbal, Arshad Jamal, Shahid Ullah, Kaffayatullah Khan, Abdullah M. Abu-Arab, Qasem M. S. Al-Ahmad, Sikandar Khan

**Affiliations:** 1 Department of Civil and Environmental Engineering, College of Engineering, King Faisal University, Al-Hofuf 31982, Saudi Arabia; kkhan@kfu.edu.sa (K.K.); 219041496@student.kfu.edu.sa (A.M.A.-A.); 219008505@student.kfu.edu.sa (Q.M.S.A.-A.); 2 Shanghai Key Laboratory for Digital Maintenance of Buildings and Infrastructure, State Key Laboratory of Ocean Engineering, School of Naval Architecture, Ocean & Civil Engineering, Shanghai Jiao Tong University, Shanghai 200240, China; mudassiriqbal29@sjtu.edu.cn; 3 Department of Civil Engineering, University of Engineering and Technology, Peshawar 25120, Pakistan; shahid.ullah@uetpeshawar.edu.pk; 4 Transportation and Traffic Engineering Department, College of Engineering, Imam Abdulrahman Bin Faisal University, Dammam 31451, Saudi Arabia; ajjamal@iau.edu.sa; 5 Department of Mechanical Engineering, King Fahd University of Petroleum & Minerals, Dhahran 31261, Saudi Arabia; sikandarkhan@kfupm.edu.sa

**Keywords:** FRP, interfacial bond strength, axial stiffness, GEP modelling, artificial intelligence, sensitivity and parametric study

## Abstract

Reinforced concrete structures are subjected to frequent maintenance and repairs due to steel reinforcement corrosion. Fiber-reinforced polymer (FRP) laminates are widely used for retrofitting beams, columns, joints, and slabs. This study investigated the non-linear capability of artificial intelligence (AI)-based gene expression programming (GEP) modelling to develop a mathematical relationship for estimating the interfacial bond strength (IBS) of FRP laminates on a concrete prism with grooves. The model was based on five input parameters, namely axial stiffness (*E_f_t_f_*), width of FRP plate (*b_f_*), concrete compressive strength (*f_c_*′), width of groove (*b_g_*), and depth of the groove (*h_g_*), and IBS was considered the target variable. Ten trials were conducted based on varying genetic parameters, namely the number of chromosomes, head size, and number of genes. The performance of the models was evaluated using the correlation coefficient (R), mean absolute error (MAE), and root mean square error (RMSE). The genetic variation revealed that optimum performance was obtained for 30 chromosomes, 11 head sizes, and 4 genes. The values of R, MAE, and RMSE were observed as 0.967, 0.782 kN, and 1.049 kN for training and 0.961, 1.027 kN, and 1.354 kN. The developed model reflected close agreement between experimental and predicted results. This implies that the developed mathematical equation was reliable in estimating IBS based on the available properties of FRPs. The sensitivity and parametric analysis showed that the axial stiffness and width of FRP are the most influential parameters in contributing to IBS.

## 1. Introduction

Reinforced concrete (RC) structures are subjected to frequent maintenance and repair due to the corrosion of conventional steel reinforcement [1].Therefore, strengthening existing structures is considered an emerging construction activity to cope with the strength requirements and upgraded code designs [2]. Fiber-reinforced polymer (FRP) laminates are widely used for retrofitting and enhancing the existing structural capacity of beams [3,4,5], columns [6,7], and beam–column joints [8,9,10] owing to their superior performance [11]. The advantages of FRP plates, such as light weight, high strength, excellent corrosion resistance [12], creep/fatigue resistance [13], and hygrothermal resistance [14], enable FRP plates to potentially replace steel plates in the application of structural reinforcement. FRP-strengthened structures are subject to variety of failures such as FRP rupture, concrete crushing, shear cracks, and debonding between concrete and laminates, which propagates through its profile, causing further damage [15,16,17]. If the ends of the strengthening plates are tightly anchored, the failure may lead to FRP rupture or crushing of the concrete [18]. Premature debonding can also be observed before reaching the ultimate capacity, among which the most frequently reported failure is debonding of the FRP laminate from one end propagating towards the centre [19]. Irreversible interfacial debonding may occur due to the diffusion of water molecules into the FRP plate, which may cause a decrease in the interlaminar shear strength of the FRP plate. The debonding may worsen with an increase in temperature [14]. The degradation of the resin due to dynamic loading and thermal aging may cause interfacial FRP debonding [20]. The debonding of FRP laminates is a critical issue that causes reduction in the structural capacity of strengthened structures [16]. A premature plate debonding from the concrete prism occurs in FRP-strengthened RC members, as observed in various experimental studies. The model of failure of a strengthened member depends on the composite action between the FRP and concrete prism [21]. If the composite action between the FRP and concrete continues, failure may ultimately come in the form of plate-end debonding and intermediate crack-induced debonding [22]. In addition, the bond’s quality due to workmanship also affects interfacial bond failures [23]. 

**Figure 1:**
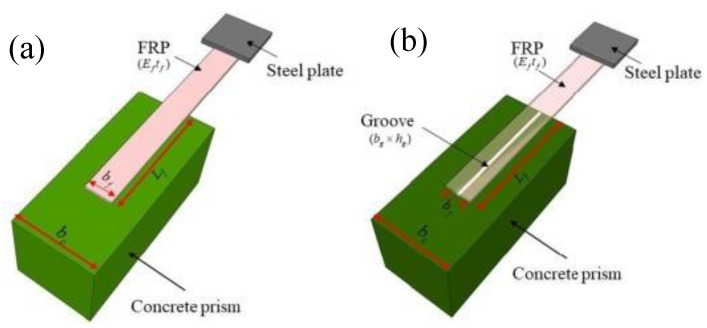
Single-lap shear test: (**a**) FRP externally bonded on concrete; (**b**) FRP externally bonded on the grooves of concrete (Reprinted/Adapted with permission from Su et al. [43]).

The existing laboratory investigations have revealed that the most important factor in premature failure in the form of FRP debonding occurs due to inappropriate preparation of the interface between the concrete and FRP [24]. Several methods, for instance, epoxy interlocking near the surface mounting, may be used to enhance the bonding between FRP and concrete [25]. Therefore, it is a desideratum to provide a suitable interface between concrete and FRP laminate for the flexural strengthening of beams. The preparation of the surface involves removing the deteriorated surface layer of the concrete and exposing coarse aggregates, thus creating better lamination. Surface treatment creates interfacial consistency between the FRP sheet and concrete surface, which leads to delayed debonding, thus increasing the ultimate rupture strength. The exposed surface of the concrete is finished by sandblasting, removing the dust using special brushes, further cleaning using solvents, and then drying before installing FRP sheets [26]. The strengthening uses direct application of FRP on concrete or near-surface mounting techniques, which comprise FRP rebars or laminates placed in the groove and then packed using high-adhesive materials. Another method involves FRP laminates externally bonded on the grooves made on the concrete’s surface (Figure 1b). The selection of suitable methods depends on the surface area, availability of material, cost, safety, and the requirement of related equipment. Among the difficulties, one can mention the rather high costs, the environmental pollution, and the facility operation steps to test the ultimate capacity of samples before their actual application. A few standard experimental procedures, such as the single-lap shear test (SST), have been used as a fundamental method to calculate interfacial bond strength (IBS), owing to its reliability and simplicity [27,28,29]. 

Empirical or semi-empirical formulations have been developed in previous studies for predicting IBS based on experimental results from SSTs [17,30,31]. The empirical relations in the proposed models fit well with the experimental data; however, these models have not been validated using new data. Moreover, basic simplified assumptions have been made in establishing these empirical relations [32]. Artificial intelligence (AI) is widely used in engineering problems in order to find the optimal solution for regression and classification problems [33,34,35,36,37,38]. AI models are not only trained using an appreciable number of experimental observations but also validated by employing a new data set [39]. In addition, AI models have many successful applications in composite structures. Vu and Hoang [40] investigated the non-linear capabilities of the least square support vector machine to predict the punching shear capacity of FRP-reinforced concrete beams in achieving the coefficient of determination (R^2^) equalling 0.99. Hoang [41] used an artificial neural network (ANN) for predicting the punching shear capacity of steel-fiber-reinforced concrete slabs. Abuodeh, et al. [42], investigated the behavior of RC beams in terms of shear capacity using a neural interpretation diagram (NID) and recursive feature elimination (RFE) algorithm.

In summary, AI models based on available experimental results are needed to predict the IBS of FRP plates on a concrete prism in order to enhance the cost-effectiveness of the engineering projects. Su, et al. [43], employed three AI models, namely, multilinear regression, support vector machine, and ANN, to predict the IBS of FRP laminates to the concrete prism. An accuracy of R^2^ equalling 0.81 and 0.91 was observed for the training and validation data, respectively. The authors opine that the developed models can be further improved in terms of accuracy. In addition, gene expression programming (GEP) is a robust technique used to establish the relationship between input and output attributes in the form of a simple mathematical equation [44]. It is noteworthy to mention that the developed ANN models are in the form of a black box. There is no information about the relationship and mathematical equation of how these attributes are related to each other. Moreover, parametric analysis is essential in order to investigate the effect of input attributes on IBS, since this may better decide which type of strengthening technique is more helpful in terms of effectiveness and economy. Therefore, the GEP model was employed to establish the mathematical relationship between the attributes and IBS of FRP laminates bonded to the concrete prism [45,46]. This research explored the capability of the GEP model in estimating the IBS of FRP laminates externally bonded to the concrete prism on the grooves using 133 experimental SST results (anchorage made on one end of FRP to the concrete prism shown in Figure 1b). Tested samples with FRP plates parallel to the groove direction were used in the analysis. The parametric analysis was also presented to see the contribution of input variables to IBS. 

## 2. Methodology

This section discusses the detailed methodology adopted for the estimation of the IBS of FRP laminates externally bonded to the concrete prism through grooves. An experimental database is explained, followed by an overview of the GEP model and its modeling procedure. The evaluation criteria for the developed models are also presented herein. 

### 2.1. Experimental Database

**Figure 2:**
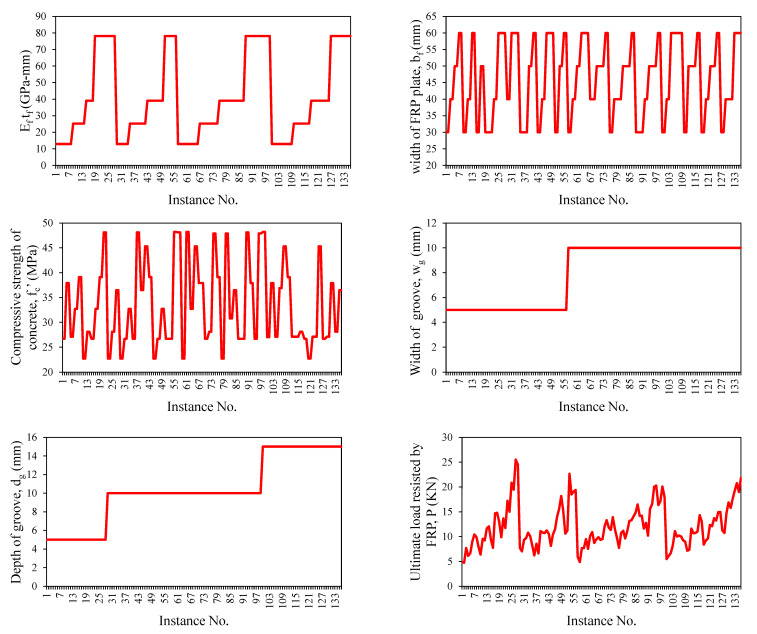
Details of variables used in the development of models.

Figure 2 illustrates the magnitude of the input and target variables used in the study. The input variables were the elastic modulus of FRP times the thickness of fiber (*E_f_t_f_*_,_ GPa-mm), which is also termed as axial stiffness, the width of the FRP plate (*b_f_*, mm), the concrete compressive strength (*f_c_*′, MPa), the width of the groove (*b_g_*, mm), and the depth of the groove (*h_g_*, mm), whereas the ultimate capacity (P, kN) was considered a target variable. The database comprised 133 experimental results of single-lap shear tests (SSTs) taken from the previous study [47], which was reported by [43]. The data were evenly distributed between the extremes. *E_f_t_f_* ranged between 12.90 to 78.90 with a skewness of 0.58. The database used in our study was experimentally conducted by Moghaddas, et al. [47]. Four different widths of FRP sheets (*b_f_* as shown in Figure 1b), equalling 30, 40, 50, and 60 mm were used in the investigation. Four variable groove sizes (5 × 5, 5 × 10, 10 × 10, and 10 × 15) were considered. Three different mix designs with concrete strengths of 25, 35, or 45 MPa were used to manifest change in the strength of concrete. The SST tests were conducted on FRP plates bonded on one side with a concrete cylinder (150 × 150 × 350 mm), as shown in Figure 1b. It is worth mentioning that the FRP surface roughness may affect IBS; however, this study was based on experimental tests conducted on FRP sheets made of the Sika wrap-200C, Sika wrap-300C, and Sika wrap-430G types of carbon and glass fibers bonded with epoxy Sikadur 330 adhesive material, as reported in Moghaddas, et al. [47]. Other statistics of the employed database are listed in Table 1. 

### 2.2. GEP Modelling 

**Figure 3:**
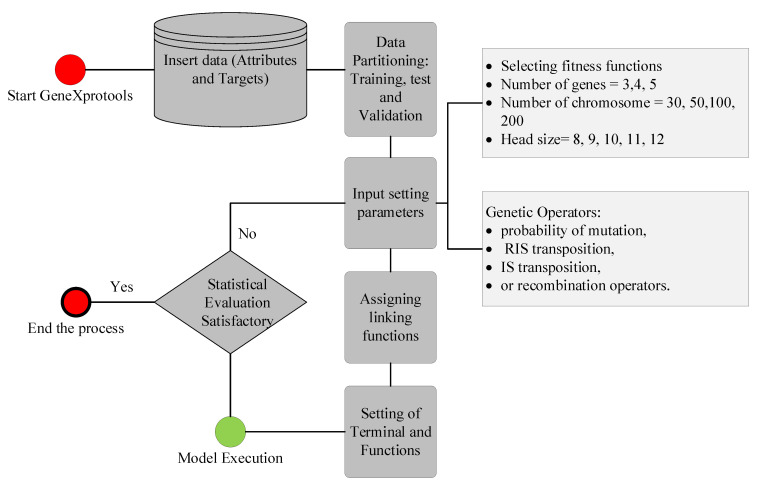
Flowchart of GEP modelling.

The GEP model, based on Darwinian principles, was inspired by the recombination of genetic materials in living organisms. An AI-based GEP model is a type of evolutionary algorithm that comprises complex trees called expression trees (ETs). The shape and size of these ETs was adjusted with the learning of the GEP model. The modelling of the GEP was carried out using GeneXproTools Version 5. Initially, the data comprising 133 data points were fed into the modelling environment. The variables were assigned as inputs and target variables. The data was partitioned into 70% training and 30% validation data using random partitioning. Subsequently, the setting parameters were varied in order to yield a high-performance model. The fitness function was selected as RMSE; the number of genes, chromosomes, and head size was varied. In addition, genetic parameters such as the probability of mutation, RIS transposition, IS transposition, or recombination operators were set according to the previous literature [38]. The linking functions within ETs were assigned as +, −, /, sqrt, and x^2^, whereas the linking function between ETs was assigned as an addition (+). The model was executed and allowed to train until the best fitness was achieved. The authors use the term “best fitness” to mean that the model was allowed to train until no further enhancement in the performance in terms of correlations and error indices was observed. At the same time, the performance of the validation data was also monitored in order to avoid over-fitness of the model. The model was stopped to generate mathematical equations upon achieving the best performance. The schematics of the GEP modelling are shown in Figure 3. 

### 2.3. Evaluation Criteria

The performance of the developed GEP models were evaluated using statistical functions, namely, coefficient of correlation (R), root mean square error (RMSE), and mean absolute error (MAE), which are common statistical indices used for the evaluation of AI models in accordance with the previous literature [37,48,49,50,51]. 

## 3. Results and Discussion

This section describes the results achieved from this study. The effect of changing genetic variables on the performance of the developed models is explained in detail, followed by the performance of the developed models. Finally, parametric and sensitivity analysis (SA) is also discussed to see the relative impact of contributing variables on interfacial bond strength. 

### 3.1. Effect of Genetic Variables 

**Figure 4:**
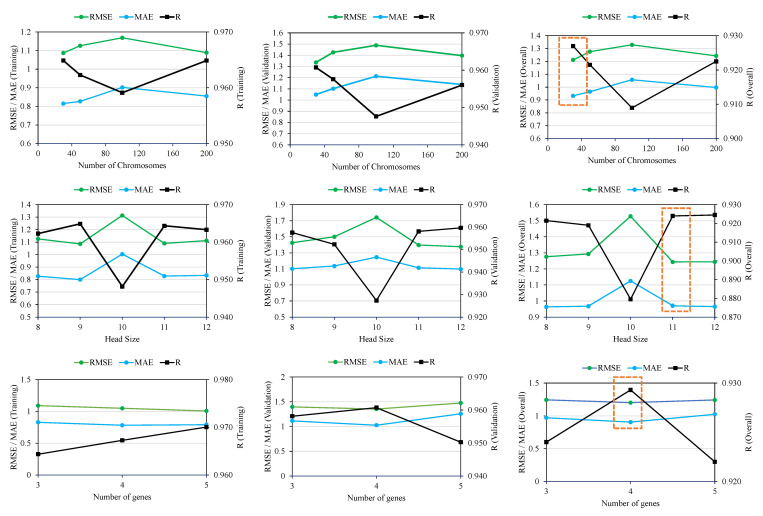
Effect of variable genetic parameters on the performance of the models (orange boxes show the optimized overall performance based on number of chromosomes, head size and genes).

**Figure 5:**
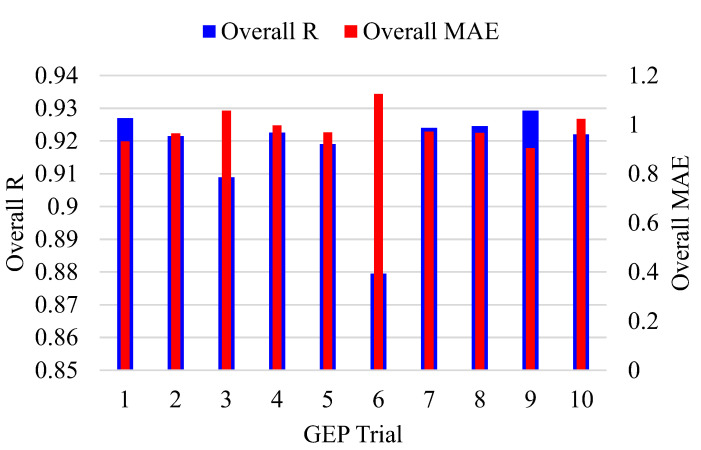
Performance indices for different trials undertaken in this study.

Figure 4 depicts the variation of genetic parameters and the corresponding change in the value of R, MAE, and RMSE. Although the performance of the models varied in the training and validation stages, the best model was selected on the basis of overall values of R, MAE, and RMSE, achieved by averaging the performance of the results of training and validation data. Initially, the number of chromosomes was varied in the order of 30, 50, 100, and 200, with constant number of genes and head size equalling 3 and 8, respectively. The values of R decreased by changing chromosomes from 30 to 50 from 0.965 to 0.962 for the training data, whereas for the validation data, R was reduced from 0.961 to 0.958 as shown in Table 2. The values of MAE and RMSE also increased with the change in number of chromosomes from 30 to 50. Further increases in chromosomes from 50 to 100 and 200 did not improve the performance of the models. The overall best performance of the models for 4 variable numbers of chromosomes was attained at a magnitude of 30. Therefore, in onward trials, chromosomes were retained at 30, and the head size was changed from 8 to 9, 10, 11, and 12. The performance of the models increased by changing head size from 8 to 9 and then decreased at 10, whereas the most optimized results were obtained at a head size of 11. This way, two parameters, i.e., number of chromosomes and head size, were optimized at 30 and 11, respectively, as tuning parameters for the next trials to be executed with a variable number of genes. It has been observed that an increase in the number of genes complexifies the output equation and the performance of the model; however, an increasing number of genes beyond five complexifies the output equation to a greater extent [38]. Figure 4 shows that four genes yielded the optimized performance of the models. In this study, 30 chromosomes, 11 head size, and 4 number of genes yielded the best performance. Previously, it has been evaluated that the optimized performance was achieved at different setting parameters [52] for different types of problems. Since it was concluded that the setting parameters generally depend on the trial and access methods in the GEP modelling, they must therefore be determined on the basis of rigorous exercise by varying the genetic parameters. Figure 5 shows that the best trial observed was trial number 9, for which the magnitude of overall R and MAE were recorded as 0.964 and 0.9045. 

### 3.2. Performance of the Developed Models

The performance of the models is presented in the form of a statistical evaluation of the training and validation data, followed by the slope of the regression line plotted between experimental and predicted observations. In addition, the predicted/experimental ratio has also been presented to see the performance of the models. 

#### 3.2.1. Statistical Evaluation

Table 2 summarises the statistical evaluation of all the trials in the form of values of R, R^2^, MAE, and RMSE. The minimum value of R for the training data was observed as 0.948, and for the validation data, the minimum value of R was recorded as 0.860 for trial 6. The maximum values of MAE were observed as 1.004 kN for the training data of trial 6, whereas for the validation data, it was recorded as 1.257 kN for trial 10. The minimum MAE was 0.782 and 1.027 kN for trial 9, and the values of R for the best trial for the training and validation data were 0.967 and 0.961, respectively. This made the average MAE equal to 6.48% and 8.52% for the training and validation data, respectively. The values of RMSE were 1.049 and 1.354 kN for the training and validation data, respectively. The statistical evaluation of all the trials showed a close agreement between experimental and predicted results; however, the results obtained from trial 9 excelled in the performance. The model in trial 9 can be used for future prediction of IBS more reliably. 

#### 3.2.2. Comparison of Regression Slopes

**Figure 6:**
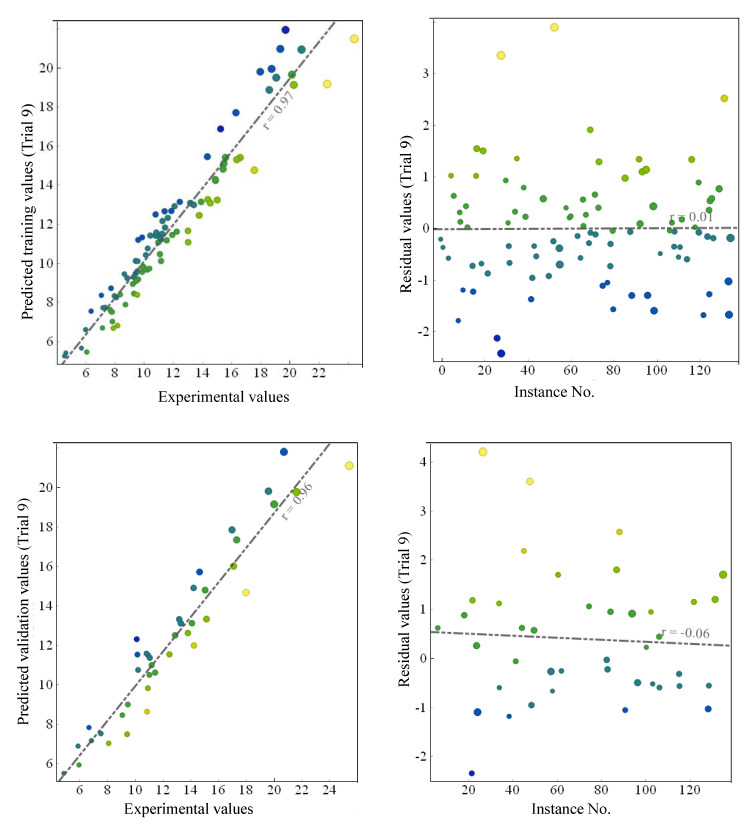
Regression slopes and error analysis of GEP model.

The regression slope of the line trending from plotting experimental results on the X-axis and predicted results on the Y-axis was investigated in this section regarding the performance of the developed models (Figure 6). A similar type of analysis in evaluating AI models has been previously practised by numerous researchers [36,37,38]. While exploring the non-linear capabilities of ANN for the compressive strength of polyethylene-terephthalate-incorporated cementitious grouts, Khan, et al. [36], found this slope equal to 1.01 and 0.90 for the training and testing data, respectively. A value of this slope more significant than 0.80 indicated agreement between the experimental and predicted results reported in the previous studies [39,51]. From Figure 6, it can be observed that the slopes for the training and validation data for trial 9 were 0.99 and 0.96, respectively. The values of the regression slopes were more significant than 0.8; therefore, the models reflected a good correlation between experimental and predicted results. Error analysis showed that the training and validation data trend line almost passed through 0 residual value. In addition, most of the residual points (experimental-predicted) lie between 1 and −1 kN. 

#### 3.2.3. Predicted to Experimental Ratio

**Figure 7:**
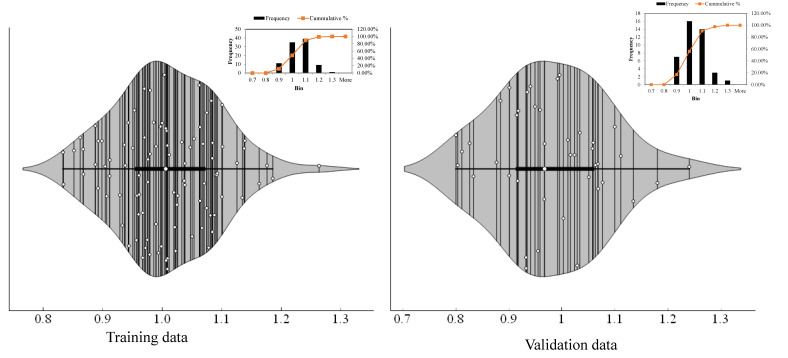
Violin plots for predicted/experimental ratio.

As discussed in Section 3.1 and Section 3.2.1, the model obtained in trial 9 was the most accurate model among the various trials investigated herein. Therefore, the results of trial 9 are plotted in the form of predicted/experimental values to manifest the accuracy in more detail. Feng, et al. [53], evaluated this ratio within ±20% while studying the XgBoost model for predicting the shear strength of squat-reinforced concrete walls. When supplemented with other statistical evaluations, the model interpreted its accuracy as higher than other empirical models. In ourstudy, Figure 7 and Table 3 show that almost 90% of data points lie between 0.9 and 1.1, which shows that the percentage of errors in predictions obtained in trial 9 are within ±10%, thus reflecting the string robustness of the developed model. This evaluation further strengthens the model for predicting IBS of FRP laminates bonded on grooves with a concrete prism.

#### 3.2.4. GEP Formulations

**Figure 8:**
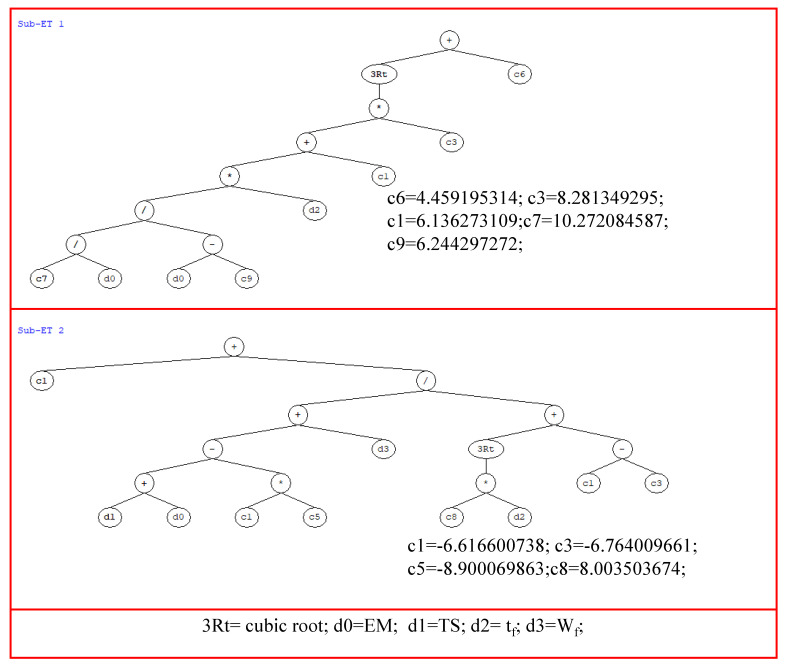

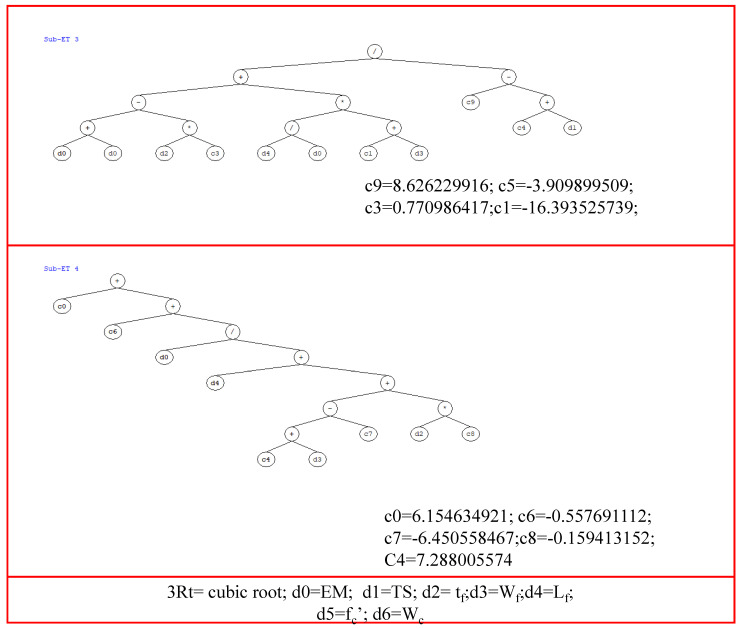
Expression trees obtained from the developed GEP model in trial 9.

The MATLAB model obtained from the GEP analysis was employed to extract simple mathematical equations for the prediction of IBS of FRP laminates bonded on grooves to the concrete prism. Figure 8 shows the ETs extracted from the GEP model, which was used to furnish the prediction equation expressed as Equation 1. It can be observed that trial 9 was executed on 4 genes; therefore, ETs contain 4 sub-ETs. The symbols denoted as c1, c2, c3, etc., among others in each sub-ET, are constants whose values are given in Figure 8. These constants and linking functions (+, −, *, /) in each sub-ET have been used to develop the mathematical equation. The following equation expressed as Equation 1 identifies that each input parameter (*E_f_* is elastic modulus of fiber; *t_f_* = thickness of fiber; *b_f_* = width of fiber plate; *f_c_*′ is concrete compressive strength; *b_g_* = width of groove; *h_g_* is depth of groove) shall be used to predict the value of IBS.

IBS = w + x + y + z(1)

where

w = ((((((10.27/*E_f_t_f_*)/(*E_f_t_f_* − 6.244)) × d(3)) − 6.14) × (−8.28))^(1/3) + 4.46);

x = (−6.62 + ((((*bf* + *E_f_t_f_*) − (−6.62(−8.90))) + b_g_)/(((8.0 *fc*′))^(1/3) + (−6.62 + 6.764))));

y = ((((*E_f_t_f_ + E_f_t_f_*) − (0.771 *fc*′)) + ((*h_g_/E_f_t_f_*) × (−16.39 + *b_g_*)))/(8.63 − (−3.91 + *b_f_*)));

z = (6.15 + (−0.56 + (*E_f_t_f_*/(*h_g_* + (((7.29 + *b_g_*) + 6.45) + (−0.159 *fc*′))))));

### 3.3. Sensitivity and Parametric Analysis

It is important to evaluate the developed models with several assessments which predict the unseen data to ensure that the prediction model possesses robustness and can forecast new data following the physical phenomenon involved in the process. Sensitivity and parametric tests demonstrate their robustness [54,55]. The SA on the simulated dataset based on the descriptive statistics of the entire database determines how susceptible a constructed model is to changes in the variables under consideration [56,57]. The relative contributions of the input factors (*E_f_t_f_, b_f_*, *f_c_*′, *b_g_,* and *h_g_*) were taken into consideration here to forecast the IBS of FRP laminates bonded to concrete with the help of grooves. This analysis was conducted on a simulated dataset created such that one first variable was varied between its extremes, and other variables were maintained at their average values. Subsequently, the second variable was varied, and so on. The predictions were made based on the trained model. For parametric analysis, the change in the value of IBS was plotted against the changing variable. For SA, Equations (2) and (3) were used. *f_max_* (*S_i_*) and *f_min_* (*S_i_*) denote, respectively, the maximum and minimum of forecasted IBS on the basis of the *i*th input domain, whereas the rest of the input variables remain constant at their mean.

(2) $$t_{i}=f_{max}\left(S_{i}\right)-f_{min}\left(S_{i}\right)$$

(3) $$SA\;\left(\%\right)=\frac{T_{i}}{\sum_{n}^{j=1}T_{j}}\times 100$$

**Figure 9:**
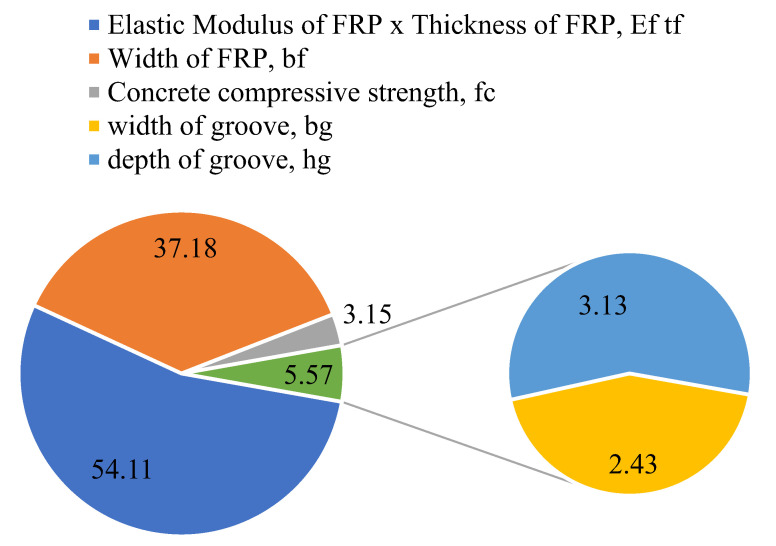
Sensitivity analysis of the developed GEP model.

**Figure 10:**
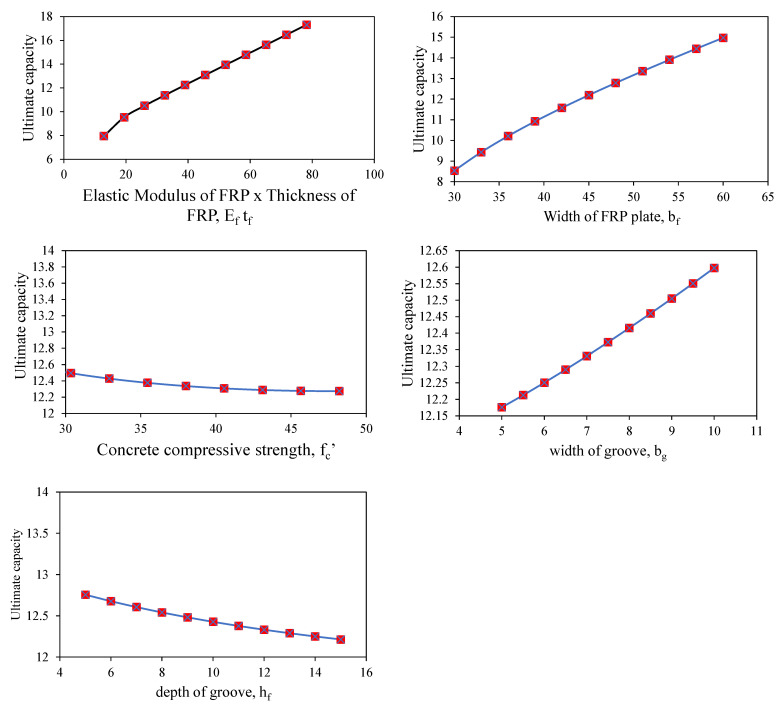
Parametric analysis of the developed GEP model.

From the Figure 9, It was observed that axial stiffness, which is termed as *E_f_t_f_* herein, had considerable influence in yielding IBS. It contributed more than 50% of the bond strength to the FRP laminates, followed by the width of FRP laminates (*b_f_*), which contributed 37.18%. The other factors, i.e., concrete compressive strength and the width and depth of the groove, contributed 8.72 altogether. This reflects that the adhesion of the FRP laminates with the groove did not considerably increase the bond strength of FRP laminates in yielding IBS. The parametric analysis in Figure 10 shows that the ultimate bond capacity linearly increased with a rise in the axial stiffness and width of the FRP laminate. The other parameters depicted no considerable change. It is important to mention that a continuous increase in these parameters would not raise the ultimate bearing capacity; however, the parametric analysis (Figure 10) was conducted to verify that the model had been trained reliably. For optimum magnitude of these parameters, a detailed study is needed based on a wide range of experiments. 

## 4. Conclusions

Due to the wide application of FRP laminates used for the retrofitting of RC elements, especially beams, columns, joints, and slabs, it is important to evaluate its bond strength with a concrete prism. For this purpose, an interfacial shear strength test was conducted on FRP laminates on a concrete prism with or without grooves to manifest interfacial bond strength (IBS). This study investigated in detail the evaluation of the important factors influencing bond strength and its prediction models employing non-linear capabilities of GEP model. The following conclusions were drawn from this study:

1. For obtaining a more robust model, ten different trials were conducted on the basis of changes in number of chromosomes, head size, and number of genes. We noticed that increasing the number of chromosomes from 30 to 200 slightly reduced the performance, whereas an 11 head size and 4 genes yielded the most accurate model (trial 9). This exercise suggests that GEP modelling requires a detailed trial and access method in order to find the optimum genetic parameters.
2. The models were evaluated using statistical indices such as R, RMSE, and MAE for both the training and validation data. The statistical indices revealed the values of R, MAE, and RMSE equalled 0.967, 0.782, and 1.049 for training and 0.961, 1.027, and 1.354 for validation, respectively. The slope of a regression line was obtained as 0.97 and 0.96 for training and validation data, respectively. This reflects a strong agreement between the experimental and predicted values. The mathematical equation based on this model has been developed to predict the interfacial bond strength of FRP laminates.
3. The sensitivity and parametric analysis showed that the axial stiffness and width of FRP were the most critical parameters in contributing to IBS. Other parameters such as concrete compression strength, width, and depth had no considerable influence in yielding IBS.

## Figures and Tables

**Table 1:** Descriptive statistics of the variables used in the development of the GEP model.

Descriptive Statistic	Input Variables					Target Variable
	Elastic Modulus of FRP × Thickness of FRP, *E_f_t_f_*	Width of FRP Plate, *b_f_*	Concrete Compressive Strength, *fc’*	Width of Groove, *b_g_*	Depth of Groove, *h_g_*	Ultimate Capacity, *P*
Unit	GPa-mm	mm	MPa	mm	mm	kN
Mean	40.33	46.10	33.72	7.94	10.33	12.05
Standard Error	2.18	1.01	0.73	0.21	0.30	0.37
Median	39.10	50.00	32.70	10.00	10.00	11.11
Mode	78.20	60.00	26.70	10.00	10.00	9.87
Standard Deviation	25.41	11.81	8.49	2.47	3.45	4.32
Sample Variance	645.42	139.52	72.15	6.10	11.93	18.65
Kurtosis	−1.23	−1.49	−1.11	−1.90	−0.88	0.30
Skewness	0.58	−0.13	0.49	−0.36	−0.09	0.80
Range	65.30	30.00	25.50	5.00	10.00	20.73
Minimum	12.90	30.00	22.70	5.00	5.00	4.76
Maximum	78.20	60.00	48.20	10.00	15.00	25.49
Sum	5484.80	6270.00	4585.40	1080.00	1405.00	1638.72
Count	136.00	136.00	136.00	136.00	136.00	136.00
Confidence Level (95.0%)	4.31	2.00	1.44	0.42	0.59	0.73

**Table 2:** Statistical evaluation of undertaken trials.

Trial No.	Used Variables	No. of Chromosomes	Head Size	Number of Genes	Constants per Gene	No. of Literals	Program Size	Training Dataset					Validation Dataset				
								Best Fitness	RMSE	MAE	R^2^	R	Best Fitness	RMSE	MAE	R^2^	R
1	4	30	8	3	10	12	40	478.96	1.087	0.815	0.931	0.965	427.920	1.336	1.049	0.923	0.961
2	4	50	8	3	10	9	39	470.33	1.126	0.827	0.926	0.962	412.230	1.426	1.102	0.917	0.958
3	3	100	8	3	10	16	43	460.85	1.169	0.902	0.920	0.959	401.750	1.489	1.213	0.898	0.948
4	4	200	8	3	10	13	39	478.65	1.089	0.855	0.931	0.965	417.150	1.397	1.139	0.914	0.956
5	4	50	9	3	10	13	42	479.17	1.086	0.800	0.931	0.965	399.420	1.500	1.136	0.907	0.952
6	4	50	10	3	10	13	43	432.1	1.314	1.004	0.899	0.948	364.750	1.742	1.246	0.860	0.927
7	4	50	11	3	10	15	48	478.32	1.090	0.828	0.930	0.964	417.180	1.397	1.114	0.918	0.958
8	4	50	12	3	10	16	54	473.45	1.112	0.834	0.928	0.963	420.810	1.376	1.098	0.921	0.960
**9**	**5**	**50**	**11**	**4**	**10**	**18**	**74**	**488.08**	**1.049**	**0.782**	**0.936**	**0.967**	**424.740**	**1.354**	**1.027**	**0.923**	**0.961**
10	5	50	11	5	10	27	92	498.05	1.007	0.791	0.941	0.970	403.840	1.476	1.257	0.903	0.950

Note: Bold numbers denotes the optimum trial.

**Table 3:** Histograms showing the frequency of each predicted/experimental ratio with a bin range of 0.10.

Training Data			Validation Data		
Bin	Frequency	Cumulative %	Bin	Frequency	Cumulative %
0.7	0	0.00%	0.7	0	0.00%
0.8	0	0.00%	0.8	0	0.00%
0.9	11	11.58%	0.9	7	17.07%
1	35	48.42%	1	16	56.10%
1.1	39	89.47%	1.1	14	90.24%
1.2	9	98.95%	1.2	3	97.56%
1.3	1	100.00%	1.3	1	100.00%
More	0	100.00%	More	0	100.00%

## Data Availability

The data used for the development of models have been reported in the paper.

## References

[1] Liberati E.A., Nogueira C.G., Leonel E.D., Chateauneuf A. (2014). Nonlinear formulation based on FEM, Mazars damage criterion and Fick’s law applied to failure assessment of reinforced concrete structures subjected to chloride ingress and reinforcements corrosion. Eng. Fail. Anal..

[2] Siddika A., Mamun M.A.A., Ferdous W., Alyousef R. (2020). Performances, challenges and opportunities in strengthening reinforced concrete structures by using FRPs—A state-of-the-art review. Eng. Fail. Anal..

[3] Yang J., Haghani R., Blanksvärd T., Lundgren K. (2021). Experimental study of FRP-strengthened concrete beams with corroded reinforcement. Constr. Build. Mater..

[4] Panahi M., Zareei S.A., Izadi A. (2021). Flexural strengthening of reinforced concrete beams through externally bonded FRP sheets and near surface mounted FRP bars. Case Stud. Constr. Mater..

[5] Kotynia R., Oller E., Marí A., Kaszubska M. (2021). Efficiency of shear strengthening of RC beams with externally bonded FRP materials–State-of-the-art in the experimental tests. Compos. Struct..

[6] Abedini M., Zhang C. (2021). Dynamic performance of concrete columns retrofitted with FRP using segment pressure technique. Compos. Struct..

[7] Hadi M.N. (2007). Behaviour of FRP strengthened concrete columns under eccentric compression loading. Compos. Struct..

[8] Tafsirojjaman T., Fawzia S., Thambiratnam D.P., Zhao X.-L. (2021). FRP strengthened SHS beam-column connection under monotonic and large-deformation cyclic loading. Thin-Walled Struct..

[9] Lee W.-T., Chiou Y.-J., Shih M. (2010). Reinforced concrete beam-column joint strengthened with carbon fiber reinforced polymer. Compos. Struct..

[10] Wu Y.-F., Jiang C. (2013). Quantification of bond-slip relationship for externally bonded FRP-to-concrete joints. J. Compos. Constr..

[11] Fathelbab F.A., Ramadan M.S., Al-Tantawy A. (2014). Strengthening of RC bridge slabs using CFRP sheets. Alex. Eng. J..

[12] Xian G., Guo R., Li C. (2022). Combined effects of sustained bending loading, water immersion and fiber hybrid mode on the mechanical properties of carbon/glass fiber reinforced polymer composite. Compos. Struct..

[13] Ding J., Cheng L., Chen X., Chen C., Liu K. (2021). A review on ultra-high cycle fatigue of CFRP. Compos. Struct..

[14] Guo R., Xian G., Li F., Li C., Hong B. (2022). Hygrothermal resistance of pultruded carbon, glass and carbon/glass hybrid fiber reinforced epoxy composites. Constr. Build. Mater..

[15] Zhang S.S., Yu T., Chen G. (2017). Reinforced concrete beams strengthened in flexure with near-surface mounted (NSM) CFRP strips: Current status and research needs. Compos. Part B Eng..

[16] Ghorbani M., Mostofinejad D., Hosseini A. (2017). Experimental investigation into bond behavior of FRP-to-concrete under mixed-mode I/II loading. Constr. Build. Mater..

[17] Sayed-Ahmed E., Bakay R., Shrive N. (2009). Bond strength of FRP laminates to concrete: State-of-the-art review. Electron. J. Struct. Eng..

[18] Teng J., Chen J.-F., Yu T. (2002). FRP-Strengthened RC Structures.

[19] Nguyen D.M., Chan T.K., Cheong H.K. (2001). Brittle failure and bond development length of CFRP-concrete beams. J. Compos. Constr..

[20] Lu Z., Xian G., Li H. (2015). Effects of exposure to elevated temperatures and subsequent immersion in water or alkaline solution on the mechanical properties of pultruded BFRP plates. Compos. Part B Eng..

[21] Bakay R.C.P. (2005). Midspan Shear Debonding of CFRP-Laminated Reinforced Concrete Beams. Master’s Thesis.

[22] Smith S.T., Teng J. (2002). FRP-strengthened RC beams. I: Review of debonding strength models. Eng. Struct..

[23] Wan B., Jiang C., Wu Y.-F. (2018). Effect of defects in externally bonded FRP reinforced concrete. Constr. Build. Mater..

[24] Chajes M.J., Finch W.W., Thomson T.A. (1996). Bond and force transfer of composite-material plates bonded to concrete. Struct. J..

[25] Jiang C., Wan B., Wu Y.-F., Omboko J. (2018). Epoxy interlocking: A novel approach to enhance FRP-to-concrete bond behavior. Constr. Build. Mater..

[26] Mostofinejad D., Mahmoudabadi E. (2010). Grooving as alternative method of surface preparation to postpone debonding of FRP laminates in concrete beams. J. Compos. Constr..

[27] Bencardino F., Condello A., Ashour A.F. (2017). Single-lap shear bond tests on Steel Reinforced Geopolymeric Matrix-concrete joints. Compos. Part B Eng..

[28] Mofrad M.H., Mostofinejad D., Hosseini A. (2019). A generic non-linear bond-slip model for CFRP composites bonded to concrete substrate using EBR and EBROG techniques. Compos. Struct..

[29] Al-Jaberi Z., Myers J.J., Chandrashekhara K. (2019). Effect of direct service temperature exposure on the bond behavior between advanced composites and CMU using NSM and EB techniques. Compos. Struct..

[30] Yuan H., Teng J., Seracino R., Wu Z., Yao J. (2004). Full-range behavior of FRP-to-concrete bonded joints. Eng. Struct..

[31] Karzad A.S., Leblouba M., Al Toubat S., Maalej M. (2019). Repair and strengthening of shear-deficient reinforced concrete beams using Carbon Fiber Reinforced Polymer. Compos. Struct..

[32] Carrara P., Ferretti D. (2013). A finite-difference model with mixed interface laws for shear tests of FRP plates bonded to concrete. Compos. Part B Eng..

[33] Bardhan A., Samui P., Ghosh K., Gandomi A.H., Bhattacharyya S. (2021). ELM-based adaptive neuro swarm intelligence techniques for predicting the California bearing ratio of soils in soaked conditions. Appl. Soft Comput..

[34] Khan M.A., Shah M.I., Javed M.F., Khan M.I., Rasheed S., El-Shorbagy M., El-Zahar E.R., Malik M. (2021). Application of random forest for modelling of surface water salinity. Ain Shams Eng. J..

[35] Khan M.A., Memon S.A., Farooq F., Javed M.F., Aslam F., Alyousef R. (2021). Compressive strength of fly-ash-based geopolymer concrete by gene expression programming and random forest. Adv. Civ. Eng..

[36] Khan M.I., Sutanto M.H., Khan K., Iqbal M., Napiah M.B., Zoorob S.E., Klemeš J.J., Bokhari A., Rafiq W. (2022). Effective use of recycled waste PET in cementitious grouts for developing sustainable semi-flexible pavement surfacing using artificial neural network. J. Clean. Prod..

[37] Iqbal M., Zhang D., Jalal F.E., Faisal Javed M. (2021). Computational AI prediction models for residual tensile strength of GFRP bars aged in the alkaline concrete environment. Ocean. Eng..

[38] Iqbal M., Zhao Q., Zhang D., Jalal F.E., Jamal A. (2021). Evaluation of tensile strength degradation of GFRP rebars in harsh alkaline conditions using non-linear genetic-based models. Mater. Struct..

[39] Jalal F.E., Xu Y., Iqbal M., Jamhiri B., Javed M.F. (2021). Predicting the compaction characteristics of expansive soils using two genetic programming-based algorithms. Transp. Geotech..

[40] Vu D.-T., Hoang N.-D. (2016). Punching shear capacity estimation of FRP-reinforced concrete slabs using a hybrid machine learning approach. Struct. Infrastruct. Eng..

[41] Hoang N.-D. (2019). Estimating punching shear capacity of steel fibre reinforced concrete slabs using sequential piecewise multiple linear regression and artificial neural network. Measurement.

[42] Abuodeh O.R., Abdalla J.A., Hawileh R.A. (2020). Prediction of shear strength and behavior of RC beams strengthened with externally bonded FRP sheets using machine learning techniques. Compos. Struct..

[43] Su M., Zhong Q., Peng H., Li S. (2021). Selected machine learning approaches for predicting the interfacial bond strength between FRPs and concrete. Constr. Build. Mater..

[44] Kaloop M.R., Samui P., Iqbal M., Hu J.W. (2022). Soft computing approaches towards tensile strength estimation of GFRP rebars subjected to alkaline-concrete environment. Case Stud. Constr. Mater..

[45] Azimi-Pour M., Eskandari-Naddaf H. (2018). ANN and GEP prediction for simultaneous effect of nano and micro silica on the compressive and flexural strength of cement mortar. Constr. Build. Mater..

[46] Faradonbeh R.S., Hasanipanah M., Amnieh H.B., Armaghani D.J., Monjezi M. (2018). Development of GP and GEP models to estimate an environmental issue induced by blasting operation. Environ. Monit. Assess..

[47] Moghaddas A., Mostofinejad D. (2019). Empirical FRP-concrete bond strength model for externally bonded reinforcement on grooves. J. Compos. Constr..

[48] Bardhan A., Gokceoglu C., Burman A., Samui P., Asteris P.G. (2021). Efficient computational techniques for predicting the California bearing ratio of soil in soaked conditions. Eng. Geol..

[49] Bardhan A., Biswas R., Kardani N., Iqbal M., Samui P., Singh M.P., Asteris P.G. (2022). A novel integrated approach of augmented grey wolf optimizer and ANN for estimating axial load carrying-capacity of concrete-filled steel tube columns. Constr. Build. Mater..

[50] Amin M.N., Iqbal M., Khan K., Qadir M.G., Shalabi F.I., Jamal A. (2022). Ensemble Tree-Based Approach towards Flexural Strength Prediction of FRP Reinforced Concrete Beams. Polymers.

[51] Jalal F.E., Xu Y., Iqbal M., Javed M.F., Jamhiri B. (2021). Predictive modeling of swell-strength of expansive soils using artificial intelligence approaches: ANN, ANFIS and GEP. J. Environ. Manag..

[52] Khan K., Jalal F.E., Iqbal M., Khan M.I., Amin M.N., Al-Faiad M.A. (2022). Predictive Modeling of Compression Strength of Waste PET/SCM Blended Cementitious Grout Using Gene Expression Programming. Materials.

[53] Feng D.-C., Wang W.-J., Mangalathu S., Taciroglu E. (2021). Interpretable XGBoost-SHAP Machine-Learning Model for Shear Strength Prediction of Squat RC Walls. J. Struct. Eng..

[54] Azim I., Yang J., Javed M.F., Iqbal M.F., Mahmood Z., Wang F., Liu Q.-F. (2020). Prediction model for compressive arch action capacity of RC frame structures under column removal scenario using gene expression programming. Structures.

[55] Shah M.I., Javed M.F., Abunama T. (2020). Proposed formulation of surface water quality and modelling using gene expression, machine learning, and regression techniques. Environ. Sci. Pollut. Res..

[56] Hanandeh S., Ardah A., Abu-Farsakh M. (2020). Using artificial neural network and genetics algorithm to estimate the resilient modulus for stabilized subgrade and propose new empirical formula. Transp. Geotech..

[57] Iqbal M.F., Liu Q., Azim I., Zhu X., Yang J., Javed M.F., Rauf M. (2020). Prediction of mechanical properties of green concrete incorporating waste foundry sand based on gene expression programming. J. Hazard. Mater..

